# Assessment of Plasma and Cerebrospinal Fluid Biomarkers in Patients with Alzheimer’s Disease and Other Dementias: A Center-Based Study

**DOI:** 10.3390/ijms26094308

**Published:** 2025-05-01

**Authors:** Francesca De Rino, Francesca Rispoli, Marta Zuffi, Eleonora Matteucci, Armando Gavazzi, Michela Salvatici, Delia Francesca Sansico, Giulia Pollaroli, Lorenzo Drago

**Affiliations:** 1 Neurology Unit, Castellanza Hospital, IRCCS MultiMedica, 20153 Milan, Italy; francesca.derino@multimedica.it (F.D.R.); marta.zuffi@multimedica.it (M.Z.); armando.gavazzi@multimedica.it (A.G.); 2 UOC Laboratory of Clinical Medicine with Specialized Areas, IRCCS MultiMedica, 20138 Milan, Italy; francesca.rispoli@multimedica.it (F.R.); elonora.matteucci@multimedica.it (E.M.); michela.salvatici@multimedica.it (M.S.); deliafrancesca.sansico@multimedica.it (D.F.S.); giulia.pollaroli@multimedica.it (G.P.); 3 Clinical Microbiology and Microbiome Laboratory, Department of Biomedical Sciences for Health, University of Milan, 20133 Milan, Italy

**Keywords:** Alzheimer’s disease, amyloid β (Aβ), hyperphosphorylated tau (pTau), mild cognitive and vascular impairment, plasma, CSF

## Abstract

Neuropsychological interviews and neuroimaging techniques are traditional diagnostic methods for Alzheimer’s disease (AD). However, the development of blood-based biomarkers, such as Amyloid beta (Aβ), phosphorylated Tau (pTau), and their ratios, offers promising non-invasive alternatives for early AD detection. This study aimed to analyze the correlation between CSF and plasma biomarkers (Aβ40, Aβ42, Aβ42/Aβ40, pTau181) and evaluate their diagnostic performance in 51 patients with cognitive impairments. Biomarkers were analyzed in both plasma and CSF using an automated chemiluminescence enzyme immunoassay, Lumipulse (Fujirebio). The results showed significant positive correlations between CSF and plasma levels of Aβ42, the Aβ42/Aβ40 ratio, and pTau181, but not for Aβ40. Plasma Aβ42, pTau181, Aβ42/Aβ40 ratio, and pTau181/Aβ42 ratio demonstrated significant differences between patients A+ vs. A− classified based on CSF Amyloid status, as well as between those classified as A+T+ and A−T− according to both CSF Amyloid and Tau levels. Plasma pTau181, Aβ42/Aβ40, and pTau181/Aβ42 ratio showed high diagnostic accuracy in distinguishing A+ from A− (AUC = 0.93–0.95) and A+T+ from A−T− patients (AUC = 0.93–0.97). These findings suggest that plasma biomarkers can effectively differentiate between AD and other forms of dementia, and serve as a reliable, non-invasive tool for early detection and monitoring of Alzheimer’s disease.

## 1. Introduction

Alzheimer’s disease (AD) is a progressive neurodegenerative disorder and the primary cause of dementia globally [1]. As the prevalence of AD continues to rise with an aging population, the disease poses considerable challenges not only to healthcare systems but also to society at large. Managing Alzheimer’s disease requires early detection, accurate diagnosis, and timely intervention to slow disease progression and improve patient outcomes. However, despite ongoing research into novel diagnostic tools, the complexities of AD and its diverse clinical presentations complicate efforts for widespread early diagnosis. Early and precise diagnosis of Alzheimer’s disease is crucial for optimizing patient care and advancing therapeutic strategies. Identifying AD in its early stages can enable clinicians to implement interventions that may help slow cognitive decline, improve quality of life, and assist in clinical trial enrolment for the development of new treatments [2,3]. However, current diagnostic methods for AD largely depend on neuroimaging techniques such as MRI and PET scans, as well as cerebrospinal fluid (CSF) biomarkers, including amyloid-beta and tau proteins [4]. CSF biomarkers have been employed as a valuable tool in the differentiation among distinct groups of neurodegenerative dementias, which often manifest common clinical symptoms, despite their differential etiology. Indeed, in most non-AD dementias, such as vascular dementia (VAD) and frontotemporal dementia (FTD), the levels of CSF biomarkers classically associated with AD (Aβ42 and tau proteins) are usually normal or only mildly elevated [5,6]. While these tools provide valuable information, they are not without limitations. Neuroimaging procedures can be costly, and while CSF biomarkers are highly informative, lumbar punctures can be invasive and are not always accessible, particularly in resource-limited settings, which relegates their use to second-line diagnostics in clinical practice [7,8]. Given these constraints, clinicians in many settings, often prefer to rely on clinical assessments, neuropsychological testing, and neuroradiological data, particularly when dealing with patients exhibiting typical AD phenotypes in which clinical observation, cognitive testing, and neuroimaging can often provide sufficient insight to support a diagnosis [9,10]. The complexity of AD, with its heterogeneous symptoms and variability in progression, reinforces the need for a multifaceted diagnostic approach. As research into Alzheimer’s disease continues, it is hoped that emerging diagnostic methods will overcome some of the current limitations, making early and accurate detection more achievable for all patients. The integration of plasma biomarkers, while still limited in routine clinical practice, holds promise for enhancing diagnostic precision, especially in atypical cases or where the diagnosis is uncertain. Recent studies showed significant positive correlations between CSF and plasma Aβ and tau phosphorylated at Threonine 181 (pTau181) by using different methods [8,9,10,11,12]. However, the use of plasma biomarkers in clinical practice remains limited by the large variability among centers, mainly due to differences in analytical platforms and assay methods used, that have still not allowed the definition of universally accepted threshold values [11,12,13,14,15]. The implementation of the fully automated platform as Lumipulse (Fujirebio) with chemiluminescent enzyme immunoassays (CLEIA) specific for plasma Aβ40, Aβ42, and pTau181 could mark a significant advancement for the development of high throughput and high reproducible tests for the diagnosis of AD [16,17,18,19,20]. In this study, we aimed to establish the correlation between CSF and plasma biomarkers and to assess the diagnostic performance of plasma Aβ42, pT181, Aβ40/Aβ42, and pTau181/Aβ42 in a cohort of patients with Alzheimer diseases and other dementias, using the Lumipulse G assays.

## 2. Results

### 2.1. Plasma Aβ42, Aβ40, Aβ42/Aβ40, and pTau181 Correlation with CSF Biomarkers

**Figure 1:**
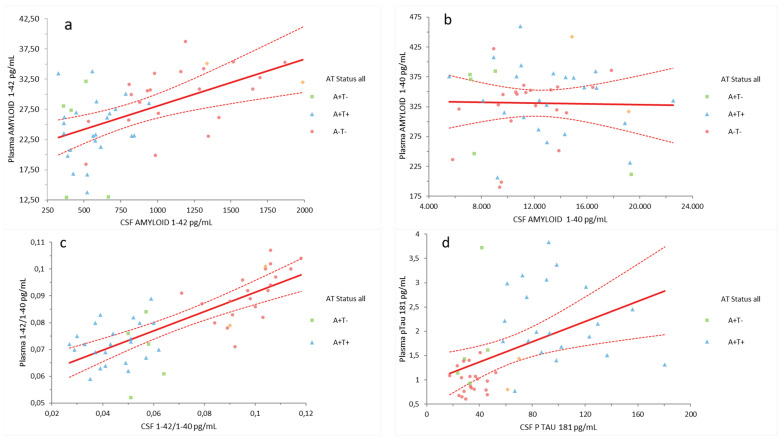
Aβ42, Aβ40, Aβ42/Aβ40, and pTau181: correlation between plasma and CSF. The diagrams represent (**a**) Aβ42 concentrations, (**b**) Aβ40 concentrations, (**c**) Aβ42/Aβ40 ratios, (**d**) pTau181 concentrations in CSF and plasma. The values expressed as pg/mL, except for Aβ42/Aβ40 ratio. Outliers: one for plasma Aβ42/Aβ40 (0.135), two for plasma pT181 (11.32 pg/mL, 5.05 pg/mL), and one for CSF pT181 (>400 pg/mL) were excluded from the analysis to enhance the accuracy of the conclusion. The symbols represent the CSF and plasma concentration of a single sample, with the colors corresponding to CSF AT status (green A+T−, blue A+T+, red A−T−). *Abbreviations*: Aβ, Amyloid beta; pTau181, tau protein phosphorylated at residue 181; CSF, cerebrospinal fluid.

Analysis of plasma and CSF biomarkers showed a weak or moderate, but significant, positive correlation for Aβ42 (r = 0.54, *p* < 0.0001) (Figure 1a), Aβ42/Aβ40 (r = 0.77, *p* < 0.0001) [Figure 1c], and pTau181 (r = 0.47, *p* = 0.0008) [Figure 1d]. Conversely, no correlation was observed between plasma and CSF Aβ40 levels [Figure 1b].

### 2.2. Plasma Biomarkers According to Amyloid and AT Classification

**Figure 2:**
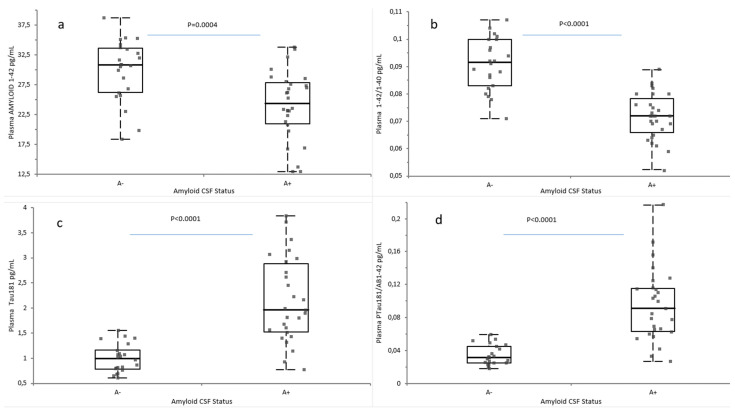
Plasma biomarkers value in A+ and A− groups. The graphs represent the box and whiskers plots of (**a**) Aβ42 concentrations, (**b**) Aβ42/Aβ40 ratios, (**c**) pTau181 concentrations, and (**d**) pTau181/Aβ42 ratio. All values are expressed in pg/mL, with the exception of the Aβ42/Aβ40 and pTau181/Aβ42 ratios. Each point corresponds to an individual value. Statistical significance was determined using a Student’s *t*-test for Aβ42 concentrations and Aβ42/Aβ40 ratios and the Mann–Whitney test for pTau181 concentrations and the pTau181/Aβ42 ratio. *Abbreviations*: Aβ, Amyloid beta; pTau181, tau protein phosphorylated at residue 181; A−, Amyloid negative; A+, Amyloid positive.

The subjects were divided into A+ (n = 28) and A− (n = 23) based on Amyloid CSF status. Plasma Aβ42/Aβ40 ratio and Aβ42, pTau181, and pT181/Aβ42 ratio levels were then analyzed in the two groups. Aβ42 was significantly lower in A+ compared to A− patients (*p* = 0.0004) [Figure 2a]. Aβ42/Aβ40 ratio was significantly lower in A+ compared to A− patients (*p* < 0.0001) [Figure 2b]. Conversely, pTau181 and pTau181/Aβ42 ratios were significantly higher in A+ compared to A− patients (*p* < 0.0001) [Figure 2c,d].

**Figure 3:**
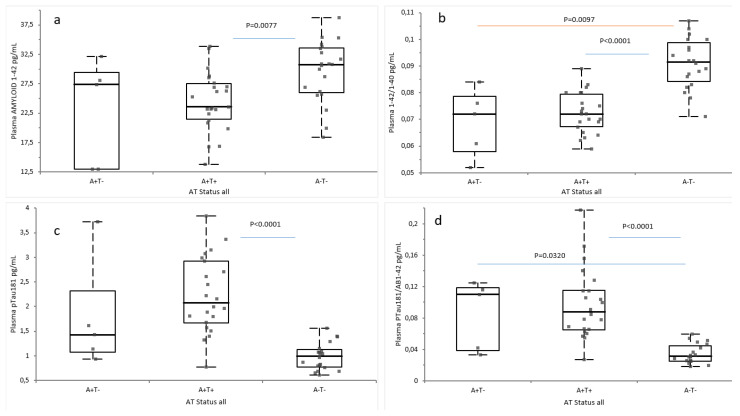
Plasma biomarkers value according to CSF status. The graphs represent the box and whiskers plots of (**a**) Aβ42 concentrations, (**b**) Aβ42/Aβ40 ratios, (**c**) pTau181 concentrations, and (**d**) pT181/Aβ42 ratios. All values are expressed in pg/mL, with the exception of the Aβ42/Aβ40 and pTau181/Aβ42 ratios. Each point corresponds to an individual value. Significant differences were assessed using a Kruskal–Wallis test followed by Steel–Dwass–Critchlow–Fligner test: n = 23, A+T+; n = 21, A−T−; n = 5, A+T−. *Abbreviations*: Aβ, Amyloid beta; pTau181, tau protein phosphorylated at residue 181; A−, Amyloid negative; A+, Amyloid positive; T−, tau negative, T+, tau positive.

In a second analysis, subjects were divided in A+T- (n = 5), A+T+ (n = 23), and A−T− (n = 21) based on CSF Amyloid and tau levels. Plasma Aβ42 was significantly decreased in A+T+ (*p* = 0.0077) compared to A−T− [Figure 3a]. Aβ42/Aβ40 was significantly decreased in A+T+ and A+T− compared to A−T− (*p* < 0.0001 and *p* = 0.0097, respectively) [Figure 3b]. Plasma pTau181 was significantly increased in A+T+ compared to A−T− (*p* < 0.0001 [Figure 3c]. pTau181/Aβ42 was significantly increased in A+T+ compared to A−T− (*p* < 0.0001) and in A+T− compared to A−T− (*p* = 0.0320) [Figure 3d].

**Figure 4:**
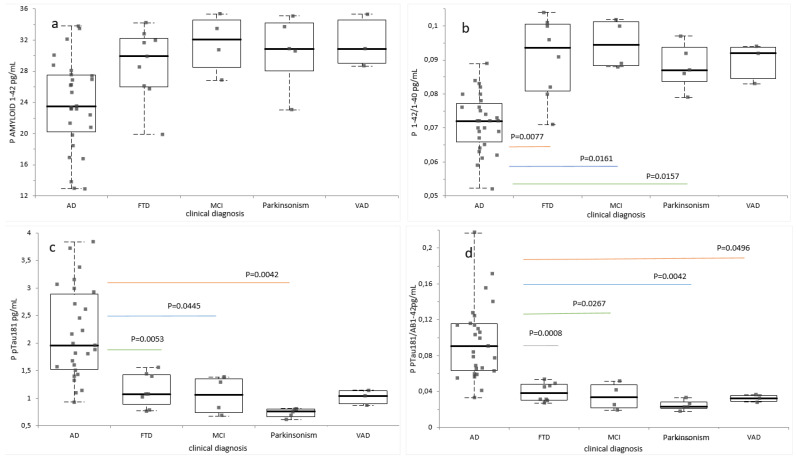
Plasma biomarkers value according to clinical diagnosis status. The graphs represent the box and whiskers plots of (**a**) Aβ42 concentrations, (**b**) Aβ42/Aβ40 ratios, (**c**) pTau181 concentrations, and (**d**) pT181/Aβ42 ratios. All values are expressed in pg/mL, with the exception of the Aβ42/Aβ40 and pTau181/Aβ42 ratios. Each point corresponds to an individual value. Significant differences were assessed using the Kruskal–Wallis test followed by Steel–Dwass–Critchlow–Fligner test: n = 28 AD; n = 9 FTD; n = 4 MCI; n = 3 VAD; n = 5 Parkinsonism. *Abbreviations*: Aβ, Amyloid beta; pTau181, tau protein phosphorylated at residue 181.

In a third analysis, the subjects were divided by clinical diagnosis: Alzheimer disease (AD), frontotemporal dementia (FTD), mild cognitive impairment [MCI], vascular dementia [VAD], and Parkinsonism [4]. Plasma Aβ42 was not significantly decreased between groups [Figure 4a]. Aβ42/Aβ40 was significantly decreased in AD compared to Other, MCI, and FTD [*p* = 0.0157, *p* = 0.0161 and *p* = 0.0077, respectively] [Figure 4b]. Plasma pTau181 was significantly increased in AD compared to FTD, Other, and MCI (*p* = 0.0053, *p* = 0.0042, and *p* = 0.0445) [Figure 4c]. pTau181/Aβ42 was significantly increased in AD compared to FTD, MCI, Other, and VAD (*p* = 0.0008, *p* = 0.0267, *p* = 0.0042, and *p* = 0.0496) [Figure 4d].

### 2.3. Diagnostic Performance of Plasma Biomarkers

**Figure 5:**
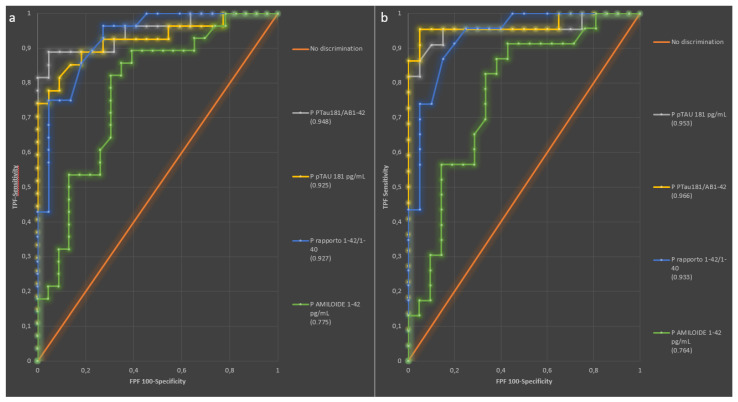
Diagnostic performance of plasma biomarker. ROC curves describing the ability of plasma biomarkers to discriminate between (**a**) A+ from A− and (**b**) A+T+ from A−T− subjects. Each ROC curve is quantified by its AUC, shown in parentheses for the corresponding biomarker. *Abbreviations*: Aβ, Amyloid beta; pTau181, tau protein phosphorylated at residue 181; ROC, receiver operating characteristic, AUC, area under the curve.

To assess the diagnostic performance of plasma Aβ42, pTau181, Aβ42/Aβ40, and pTau181/Aβ42 in distinguishing A+ from A− and A+T+ from A−T− belonging to Neurodegenerative disorder patients, a ROC curve analysis was performed. When comparing A+ and A− patients, the AUCs were 0.778 (95% CI 0.645–0.910) for Aβ42, 0.924 (95% CI 0.0.854–0.0.994) for Aβ42/Aβ40, 0.925 (95% CI 0.850–1.000) for pTau181, and 0.948 (95% CI 0.888–1.008) for pTau181/Aβ42 [Figure 5a]. The optimal thresholds were 28.68 pg/mL (sensitivity 82.1%, specificity 69.6%) for Aβ42, 0.078 (sensitivity 75.0%, specificity 95.5%) for Aβ42/Aβ40, 1.56 pg/mL (sensitivity 74.1%, specificity 100%) for pTau181, and 0.054 (sensitivity 88.9%, specificity 95.5%) for pTau181/Aβ42.

Successively comparing A+T+ and A−T− subjects, the AUC was 0.768 (95% CI 0.622–0.915) for Aβ42, 0.930 (95% CI 0.857–1.002) for Aβ42/Aβ40, 0.953 (95% CI 0.883–1.023) for pTau181, and 0.966 (95% CI 0.906–1.025) for pTau181/Aβ42 (Figure 5b). The optimal thresholds were 28.68 pg/mL (sensitivity 82.6%, specificity 66.7%) for Aβ42, 0.082 (sensitivity 87.0%, specificity 85.0%) for Aβ42/Aβ40, 1.56 pg/mL (sensitivity 81.8%, specificity 100%) for pTau181, and 0.054 (sensitivity 95.5%, specificity 95.0%) for pTau181/Aβ42.

## 3. Discussion

The measurement of neurodegeneration biomarkers, Aβ42, T-tau, and p-tau181, play a crucial role in the diagnosis of Alzheimer’s disease and other dementias. In fact, a variation in CSF concentrations of these proteins provides, in vivo, evidence of the histopathological modifications characteristic of the disease, such as the development of extracellular plaques of β-amyloid and neurofibrillary tangles of tau and phospho-tau [7]. Moreover, the evaluation of these markers allows for discriminating forms of AD dementia from dementias of other origins and identifying those forms of mild cognitive deterioration that will evolve into AD dementia [5,6]. CSF biomarker analysis has proven to be essential for detecting early signs of AD, so much so that their measurement, together with topographic markers, has been included in the recommendations of the International Working Group (IWG) and the National Institute on Aging—Alzheimer’s Association—for the diagnosis of AD [21]. Although CSF biomarkers offer valuable insights into the molecular changes associated with Alzheimer’s disease, aiding early diagnosis, tracking disease progression, and supporting clinical decision-making, the lumbar punctures required for CSF collection remain invasive, uncomfortable, and not always accessible to all patients. Plasma biomarkers represent a promising alternative that could overcome some of the limitations associated with CSF analysis. Plasma collection is non-invasive, cost-effective, and easier to implement on a larger scale, making it a more practical approach for routine clinical use [22]. Plasma biomarkers present a more accessible alternative for monitoring AD progression, especially in cases where repeated testing is necessary or when patients are unable or unwilling to undergo lumbar punctures. Advances in blood-based biomarker research have identified plasma markers, such as Amyloid-beta, tau, and neurofilament light chain (NfL), which strongly correlate with CSF findings and brain-imaging results. Recent technological advancements have significantly enhanced their sensitivity and specificity, positioning blood-based biomarkers as a promising frontier in the diagnosis and management of AD and related dementias [23]. Their potential for widespread use could significantly reduce the burden on healthcare systems and improve early detection of AD in a more cost-effective and patient-friendly manner. In the present study, we evaluated the ability of plasma biomarkers to reflect CSF profile and assessed the diagnostic performance of plasma Aβ40, Aβ42, Aβ42/Aβ40, pTau181, and pTau181/Aβ42 in a cohort of patients with Alzheimer’s disease and other dementias, using the chemiluminescence-based Lumipulse G assay platform.

Our results showed a significant positive correlation between CSF and plasma for Aβ42, pTau181, and for Aβ42/Aβ40 ratio, while plasma Aβ40 levels did not correlate with CSF. The pAβ42/Aβ40, pT181/Aβ42, and pTau181 ratios demonstrated the strongest association with the CSF profile, suggesting their potential as valuable plasma biomarkers for predicting CSF characteristics. These findings are consistent with recent research [16,17,18,19,20,21,22,23,24]. Wilson et al. [24], in a 2022 study evaluating the performance of the Lumipulse assay for Alzheimer’s disease, found a positive association between plasma p-Tau181 and CSF p-Tau181 (β = 0.543, *p* < 0.001); similarly, Silva-Spinola et al. [19] found that p-Tau181 had the highest correlation between the two fluids (ρ = 0.61; *p* < 0.001), followed by the p-Tau181/Aβ42 ratio with a moderate correlation (ρ = 0.57; *p* < 0.001), while the Aβ42/Aβ40 ratio showed a weaker correlation (ρ = 0.39; *p* < 0.001). Successively, for assessment of the diagnostic performance of plasma biomarkers, we analyzed plasma biomarker levels first among A+ and A− subjects, defined by their Amyloid CSF status, then among four different CSF A/T groups based on CSF Amyloid and tau levels. The Aβ42 and Aβ42/Aβ40 ratios were significantly lower in A+ vs. A− and A+T+ vs. A−T− patients; conversely, the pTau181 and pTau181/Aβ42 ratios were significantly higher in A+ vs. A− and A+T+ vs. A−T− patients. Plasma pTau181, Aβ42/Aβ40, and pTau181/Aβ42 ratios showed high diagnostic accuracy in distinguishing A+ from A− (AUC = 0.93–0.95) and A+T+ from A−T− patients (AUC = 0.93–0.97). Our results are consistent with the results of the Bellomo et al. [16] study, which aimed to analyze the diagnostic performance of AD biomarkers using plasma samples. Bellomo et al. showed that the Aβ42/Aβ40 ratio presented the best performance when A+ and A− groups were compared, whereas pTau181 was an effective biomarker in differentiating A+T+ from A−T− subjects. Plasma levels of Aβ42 and the Aβ42/40 ratio were significantly lower in A+ patients, regardless of T status, compared to A− patients, whereas plasma p-tau181 was significantly higher in A+/T+ individuals compared to all other groups. The authors did not find significant biomarker changes in the A−/T+ group versus the A−/T− group, including p-tau181, but noted some small differences in p-tau181 levels between A−/T− and A+/T− groups. The ROC analysis confirmed these findings, showing plasma p-tau181 as the best test in distinguishing A+/T+ from A−/T− and A+/T+ from other groups and the Aβ42/40 ratio as the most effective in distinguishing A+ from A− and A+/T− from A−/T−. Aβ42 alone performed worse than the Aβ42/40 ratio, and none of the biomarkers could differentiate between A−/T− and A−/T+ groups.

Finally, we tried to propose optimal thresholds for plasma Aβ42, pTau181, Aβ42/Aβ40, and pTau181/Aβ42 measurements for the best identification of CSF A+ and A+/T+ profiles. Over the past decade, significant effort has been made to enable the measurement of AD biomarkers in blood, offering clear advantages over traditional CSF-based approaches. The ultrasensitive measurement of plasma Aβ42, pTau181, and Aβ42/Aβ40 showed a satisfactory agreement with CSF AD biomarkers and a strong relationship with clinical signs. However, variability between laboratories and between assays have been observed, making difficult the definition of reliable cut-off values for the clinical use of plasma AD biomarkers, so much so that current recommendations suggest the use of internally defined cut-offs for different populations.

One of the limitations of this study is the relatively small sample size of 51 patients, which may limit the generalizability of the results to a broader population. Additionally, the cohort consisted of patients with cognitive impairment who were already indicated for second-level investigations, which may introduce selection bias and may not fully represent the general population of individuals at risk for Alzheimer’s disease.

Third, we were unable to evaluate the effects of comorbidities and risk factors associated with blood-based biomarkers (i.e., body mass index, chronic kidney disease, etc.) due to inaccessibility to pertinent information. Recent evidence suggests that the plasma concentrations of Aβ42 and pTau181 are affected by confounding factors, especially due to kidney diseases [18,23,25,26]. The use of the Amyloid ratio is very useful in overcoming this issue, since Aβ40 and Aβ42 are similarly influenced by the glomerular filtration rate, and this finding further strengthens the relevance of Aβ42/Aβ40 in predicting the AD CSF profile.

The effects of confounding factors on pTau levels are less clear, since some authors did not find significant effects [27,28,29], whereas others suggested that pTau levels are increased in patients affected by kidney diseases [16]. Another limitation is the absence of the control group: invasiveness of CSF collection dissuades participants from AD research, whereas the minimally invasive nature of plasma sampling could improve recruitment efforts. Finally, we focused on specific biomarkers (Aβ40, Aβ42, and pTau181), which, while promising, may not capture the full complexity of Alzheimer’s pathology. Therefore, although this study shows promising diagnostic performance, further validation in larger, more diverse cohorts and in clinical practice settings is needed to confirm the utility of plasma biomarkers as a non-invasive alternative for early detection and monitoring of neurodegenerative disorders.

## 4. Materials and Methods

### 4.1. Study Participants

The samples studied consist of a consecutive series of 51 patients with cognitive impairments that were referred to the Neurology Unit of Multimedica, Castellanza (Varese, Italy) from August 2022 to May 2024 and were selected for a second-level analysis, which was necessary for a more specific diagnostic framework. Plasma and cerebrospinal fluid [CSF] samples were used in this study for the measurement of Aβ40, Aβ42, and pTau181. In total, 28 were males, and 23 were females subjects. The mean age was 70 years (standard deviation, SD = 8). The patients were divided into Amyloid positive (A+) and Amyloid negative (A−) based on Amyloid CSF status (A+ = CSF Aβ42/Aβ40 < 0.069, n = 28; A− = Aβ42/Aβ40 > 0.069, n = 23). In a second analysis, the subjects were divided in Amyloid positive and tau negative (A+T−), Amyloid positive and tau positive (A+T+), Amyloid negative and tau negative (A−T−), and Amyloid negative and tau positive (A−T+) based on CSF Amyloid and tau levels (A+T− = CSF Aβ42/Aβ40 < 0.069 and pTau181 < 56.5 pg/mL, n = 5; A+T+ = CSF Aβ42/Aβ40 < 0.069 and pTau181 > 56.5 pg/mL, n = 23; A−T− = CSF Aβ42/Aβ40 > 0.069 and pTau181 < 56.5 pg/mL, n = 21; A−T+ = CSF Aβ42/Aβ40 > 0.069 and pTau181 > 56.5 pg/mL, n = 2). A description of the cohort, the distribution according to AT classification and a descriptive analysis of the CSF and plasma biomarkers are reported in Table 1.

In a third analysis, patients were divided by clinical diagnosis as follows: 28 patients with AD, 9 with frontotemporal dementia (FTD), 4 with mild cognitive impairment (MCI), 3 with vascular dementia (VAD), 2 with progressive supranuclear palsy (PSP), and 5 with Parkinsonism.

### 4.2. CSF and Plasma Sample Collection

Cerebrospinal fluid (CSF) samples were obtained in the morning via lumbar puncture at the L4-L5 level and promptly centrifuged at 2000× *g* for 10 min within a few hours of collection. A total of 800 µL of the supernatant was aliquoted into 1.5 mL polypropylene micro-tubes (Sarstedt) and stored at −80 °C for future analysis. Whole blood was drawn into BD Vacutainer EDTA tubes and centrifuged at 2000× *g* for 10 min. Following centrifugation, 800 µL of plasma was transferred into 1.5 mL polypropylene micro-tubes (Sarstedt) and stored at −80 °C. Both CSF and plasma samples were shipped on dry ice from the Neurology Department of Castellanza to the Multilab facility at Multimedica, where the biomarker analysis was conducted.

### 4.3. Measurement of CSF and Plasma Biomarkers

CSF biomarkers, including Aβ40, Aβ42, total tau, and pTau181, along with plasma biomarkers Aβ40, Aβ42, and pTau181, were quantified using a fully automated chemiluminescence enzyme immunoassay (CLEIA) on the Lumipulse G1200 system (Fujirebio), as described elsewhere [23].

Prior to the analysis, the CSF samples were thawed at room temperature for 30 min and vortexed for 10 s. Plasma samples were similarly thawed for 30 min at room temperature, vortexed for 10 s, and then centrifuged at 2000× *g* for 5 min before proceeding with the analysis.

### 4.4. Statistical Analysis

Normality was evaluated using a Kolmogorov–Smirnov test and Q-Q plots. Outliers: one for plasma Aβ42/Aβ40 (0.135), two for plasma pT181 (11.32 pg/mL, 5.05 pg/mL), and one for CSF pT181 (>400 pg/mL) were excluded from the analysis to enhance the accuracy of the conclusion. The Student’s *t* test or the Mann–Whitney test were used to compare plasma biomarkers in A+ and A− subjects. The Kruskal–Wallis test, followed by the Steel–Dwass–Critchlow–Fligner test, was used to make all possible pairwise comparisons across the three groups A+T+, A+T−, and A−T− and clinical diagnosis groups (AD, FTD, MCI, VAD, and Other). A−T+ and PSP were excluded from the analyses since they consist of only two values and could have led to less precise results. The correlations between the CSF and plasma Aβ42/Aβ40 ratio, pTau181, Aβ42, and Aβ40 levels were visualized using scatter plots and analyzed with Pearson’s correlation coefficient. The diagnostic performance of plasma Aβ42, Aβ42/Aβ40, pTau181, and pT181/Aβ42 in distinguishing A+ from A− and A+T+ from A−T− patients was analyzed by means of a receiver operating characteristic (ROC) curve analysis using a Wilcoxon–Mann–Witney AUC estimator. To compare the ROC curves, the area under the curve (AUC) was considered. The thresholds were determined by maximizing the Youden index, and sensitivity and specificity were calculated for each biomarker. The statistical analysis was performed using Excel Analyse-it ^®^ v. 6.15.4 (Leeds, UK), and a pROC package in R was used for the ROC analysis. *p*-values < 0.05 were considered significant [* < 0.05; ** < 0.01; *** < 0.001; **** < 0.0001].

## 5. Conclusions

This study aims to explore the diagnostic potential of serum biomarkers in Alzheimer’s disease, comparing them with the liquor data and focusing on their ability to detect early pathological changes and differentiate AD from other neurodegenerative conditions. By integrating clinical data with biomarker analysis, we seek to contribute to the development of accessible and reliable tools for the early diagnosis and management of Alzheimer’s disease.

Overall, our results suggest the utilization of plasma biomarkers as a non-invasive and cost-effective tool to support AD diagnosis. Among them, plasma Aβ42/Aβ40, pT181/Aβ42, and pT181 show the best diagnostic performances, and, for this reason, they seem the most promising candidates for the replacement of CSF biomarkers in the detection of AD pathology, especially in the prodromal phases of the disease.

There remains the risk of having an “overly simplified” tool for such a dramatic diagnosis without real certainties. For this reason, it will be necessary to continue identifying even more truthful and specific biomarkers (PTAU 217). Our study has further demonstrated the reliability of plasma AD biomarkers as an economic and non-invasive tool to improve the management of patients with neurodegenerative disorders. This additional study has indeed tried to confirm and clarify this complicated puzzle, with a significant contribution of information provided by the laboratory in order to improve patient’s management.

## Figures and Tables

**Table 1:** Sample description.

	n = 51
**Characteristics**	
Males, n [%]	28 [55%]
Age, mean ± SD	70 ± 8
**Diagnosis**	
AD	28 [55%]
FTD	9 [17%]
MCI	4 [8%]
VAD	3 [6%]
PSP	2 [4%]
Parkinsonism	5 [10%]
**CSF biomarkers**	
Aβ42, mean ± SD, pg/mL	831 ± 427
Aβ40, mean ± SD, pg/mL	12,301 ± 3867
Aβ42/Aβ40, mean ± SD	0.069 ± 0.028
Total tau, mean ± SD, pg/mL	469 ± 319
pTau 181, mean ± SD, pg/mL	71.7 ± 62.8
**Plasma biomarkers**	
Aβ42, mean ± SD, pg/mL	26.77 ± 6.16
Aβ40, mean ± SD, pg/mL	331.07 ± 61.25
Aβ42/Aβ40, mean ± SD	0.081 ± 0.015
pTau 181, mean ± SD, pg/mL	1.89 ± 1.66
pTau 181/Aβ42, mean ± SD	0.077 ± 0.070
**A classification, n [%]**	
A+	28 [55%]
A−	23 [45%]
**AT classification, n [%]**	
A+T−	5 [10%]
A+T+	23 [45%]
A−T−	21 [41%]
A-T+	2 [4%]

*Abbreviations*: SD: standard deviation; Aβ: Amyloid beta; pTau181: tau protein phosphorylated at residue 181; A−: Amyloid negative; A+: Amyloid positive; T−: tau negative: T+: tau positive. (AD): Alzheimer Disease; (FTD): frontotemporal dementia; (MCI): mild cognitive impairment; (VAD): vascular dementia; (PSP): progressive supranuclear palsy.

## Data Availability

Raw data are available upon reasonable request. The requests should be addressed to the corresponding author.

## List of Abbreviations

Aβ	Amyloid β
AD	Alzheimer’s Disease
AUC	Area Under the Curve
CLEIA	ChemiLuminescent Enzyme ImmunoAssay
CI	Confidence Interval
CSF	Cerebrospinal Fluid
EDTA	EthyleneDiamineTetraacetic Acid
IQR	Interquartile Range
KD	Kidney Disease
MCI	Mild Cognitive Impairment
NIA-AA	National Institute on Aging and Alzheimer’s Association
pTau	Hyperphosphorilated tau
ROC	Receiver Operating Characteristic
SD	Standard Deviation
